# Novel Blood-Biomarkers to Detect Retinal Neurodegeneration and Inflammation in Diabetic Retinopathy

**DOI:** 10.3390/ijms26062625

**Published:** 2025-03-14

**Authors:** Javad Nouri Hajari, Tomas Ilginis, Tobias Torp Pedersen, Claes Sepstrup Lønkvist, Jon Peiter Saunte, Mikael Hofsli, Diana Chabane Schmidt, Hajer Ahmad Al-abaiji, Yasmeen Ahmed, Daniella Bach-Holm, Line Kessel, Miriam Kolko, Mette Bertelsen, Lars Michael Larsen, Frederik Sørensen, Julie Lyng Forman, Dorte Aalund Olsen, Thomas Rosenberg, Ivan Brandslund, Carina Slidsborg

**Affiliations:** 1 Department of Ophthalmology, Rigshospitalet, 2600 Glostrup, Denmark; javad.nouri.hajari@regionh.dk (J.N.H.); tomas.ilginis@regionh.dk (T.I.); tobias.emil.torp-pedersen@regionh.dk (T.T.P.); claes.sepstrup.loenkvist.02@regionh.dk (C.S.L.); jon.peiter.saunte@regionh.dk (J.P.S.); mikael.hofsli@regionh.dk (M.H.); diana.chabane.schmidt.01@regionh.dk (D.C.S.); hajer.ahmad.al-abaiji@regionh.dk (H.A.A.-a.); zkp853@alumni.ku.dk (Y.A.); daniella.bach-holm.01@regionh.dk (D.B.-H.); line.kessel.01@regionh.dk (L.K.); miriam.kolko@regionh.dk (M.K.); lars.michael.larsen@regionh.dk (L.M.L.); tro@eyenet.dk (T.R.); 2 Department of Clinical Medicine, University of Copenhagen, 2200 Copenhagen, Denmark; 3 Department of Clinical Genetics, Rigshospitalet, 2200 Copenhagen, Denmark; mette.bertelsen.vardrup@regionh.dk; 4 Section of Biostatistics, Department of Public Health, University of Copenhagen, 1353 Copenhagen, Denmark; fryggy@gmail.com (F.S.); jufo@sund.ku.dk (J.L.F.); 5 Department of Biochemistry and Immunology, University of Southern Denmark, Vejle Hospital, 7100 Vejle, Denmark; dorte.aalund.olsen@rsyd.dk (D.A.O.); ivan.brandslund@rsyd.dk (I.B.)

**Keywords:** plasma-biomarkers, chronic retinal degenerative disease, diagnosis, disease monitoring, confounders, age, medical comorbidities, diabetic microvascular complication

## Abstract

To investigate levels of specific plasma-biomarkers related to neurodegeneration and inflammation in patients with different chronic degenerative retinal diseases, using an ultrasensitive technology called ‘single molecule array’ (SiMoA). Also, to investigate if biomarkers were measurable in the patient’s blood, dependent on age and medical comorbidities, and useful for stratifying the diseases. This exploratory, cross-sectional study recruited 151 adults at the Department of Ophthalmology, Rigshospitalet, Denmark (period 2019 to 2020). Clinical data came from the electronic medical-record system. The study population consisted of 131 patients: 32 with diabetic retinopathy (DR; 51 diabetes, DM), 27 with glaucoma, 53 with inherited retinal degeneration (IRD and 20 healthy controls (HC). Medical comorbidities included organ failure, other active eye diseases, and comorbidities. Three biomarkers, neurofilament-light-chain (NFL), glial-fibrillary-acidic-protein (GFAP), and CXC-motif chemokine ligand 13 (CXCL13), were measured with SiMoA technology. The age-adjusted values were reported as fold differences (FD) with 95% confidence intervals (CI). Increased NFL levels were found in DR patients compared to HCs (FD 1.81 95%CI 1.43, 2.28, *p* < 0.001, adj-*p* < 0.001). Similarly increased NFL levels were reported in advanced DR (PDR, DME), compared to both DM (FD 2.52 (95%CI: 1.71; 3.72, *p* < 0.001, adj-*p* < 0.001, and FD 2.04 (95%CI: 1.33; 3.12, *p* < 0.001, adj-*p* < 0.001), respectively) and HCs (FD 2.35 (95%CI: 1.67; 3.30, *p* < 0.001, adj-*p* < 0.001), and FD 1.89 (95%CI: 1.28; 2.79, *p* < 0.001, adj-*p* < 0.001) respectively). Independent of comorbidities, decreased NFL-levels were seen in IRD compared to DR (FD 0.49 (95% CI 0.39; 0.61, *p* < 0.001; adj-*p* < 0.001), ±comorbidities). Decreased GFAP levels were seen in DM patients compared to HCs (FD 0.69; 95%CI 0.55, 0.87, *p* = 0.002, adj-*p* = 0.02), but contrary to an increasing trend in advanced DR compared to DM (-comorbidities). These results imply that these biomarker-tests are useful for detecting and monitoring development of retinopathy in the circulations of diabetes patients. Plasma-biomarkers may be useful to stratify between retinal disease types. Prospective studies are underway to explore this hypothesis in depth.

## 1. Introduction

Over decades, innovative OMICs technologies have advanced molecular profiling to reflect human disease with high precision [1]. Mass-spectrometry has been shown to be a very useful experimental tool, although detection of low-abundance molecules remains difficult. (1) Digital ELISA technique *Single Molecular Assay* (SiMoA) technology enables accurately quantifying proteins down to the femtomolar concentrations, hence the SiMoA platform has become a valuable and widely used tool to detect and monitor biomarkers for disease [2,3].

The eye is an immune-privileged organ having blood-retinal barriers to regulate movement of substances across the vessel walls. In certain chronic degenerative retinal diseases, such as diabetic retinopathy (DR), inherited retinal disease (IRD), age-related macular degeneration (AMD), and retinopathy of prematurity (ROP), a breakdown of these barriers results in the formation of retinal edema [4,5,6]. We speculate whether molecules present in a diseased retina can reach circulation. Determination of valid blood-biomarkers for retinal diseases may be valuable in detecting very early disease and allowing for monitoring of disease progression with high accuracy, either alone or in combination with a clinical eye examination program. In addition, a valid blood-biomarker may allow outpatient disease monitoring.

Neurodegeneration, inflammation, and activation of glial cells are involved in the pathogenesis of various chronic degenerative retinal diseases (including IRD, DR, glaucoma, and AMD) [7,8,9,10]. Determination of biomarkers released into circulation from the retina during disease progression would reveal potential diagnostic and prognostic biomarkers usable in a clinical care setting. In addition, the newly found biomarkers may be key drivers of disease development, and potential therapeutic targets for new treatments. Thus, identification of valid biomarkers for pathophysiological mechanisms occurring in retina is warranted. To further explore this issue, the biomarker for investigation needs to be carefully selected based on its origin and location in the body (i.e., retina, or elsewhere in the body).

In the central and periphery nervous system, the neurofilament light chain (NFL) is an integrated part of the axonal cytoskeleton securing cell structure and integrity [10]. In retinal ganglion cells (inner retinal layer), NFL is also a structural cell component. NFL is an established blood-biomarker for several neurological diseases [11,12].

The glial-fibrillary-acidic-protein (GFAP, type-III intermediate filament protein) is a structural protein localized in glia-cells in nervous tissue. In the central nervous system, GFAP molecules are found in the astrocyte cytoplasm, while in retina GFAP is found in both astrocytes and Müller cells trespassing the entire retina [13,14]. GFAP is an emerging blood-biomarker for medical disorders in the brain and spinal cord [15,16].

Recently, attention has been directed towards another biomarker for neuroinflammation: CXC-motif chemokine ligand 13 (CXCL13). It is a chemokine-ligand of the B lymphocyte receptor CXCR5, and part of the immune response expressed in several tissues, such as brain, vascular and glial cells in the retina [17,18]. This blood-biomarker has shown potential to indicate early signs of inflammation in diseases in the central nervous system [19].

Previous studies on potential blood-biomarkers to diagnose and monitor disease progression of chronic degenerative retinal diseases is sparse and the topic highly controversial, as it is unclear if and how intraretinal molecules could potentially reach blood circulation. Herein, by using the ultrasensitive SiMoA-platform, we explore blood-levels of several potential biomarkers, NFL, GFAP and CXCL13, in patients with different chronic degenerative retinal diseases. Additionally, we investigated the impact that age and other medical disorders could have on biomarker concentration. In this report we focus our results and discussions mainly on the age-adjusted findings.

## 2. Results

Clinical characteristics for study participants are presented in Table 1 according to the absence or presence of comorbidities. In the case of certain data considered not clinically important, they are missing and notified NA accordingly. The diabetes patients with DR were older than HCs. Additionally, ill DR patients (+comorbidities) had lower eGFR Hb, and HDL, along with higher Creatinine, Hb1Ac, Glucose, VLDL, Triglycerides, BP (systolic, diastolic), biothesiometry, weight, and BMI, than the otherwise healthy DR subgroups (-comorbidities). The IRD patients had reduced VA, and impacted macula-OCT measurements compared to HCs, while glaucoma patients had moderate VA reduction, more visual field deficits, abnormally low papil-OCT values, and reduced ganglion cell layer (GCL) thickness, compared to the expected values in the normal range.

**Figure 1:**
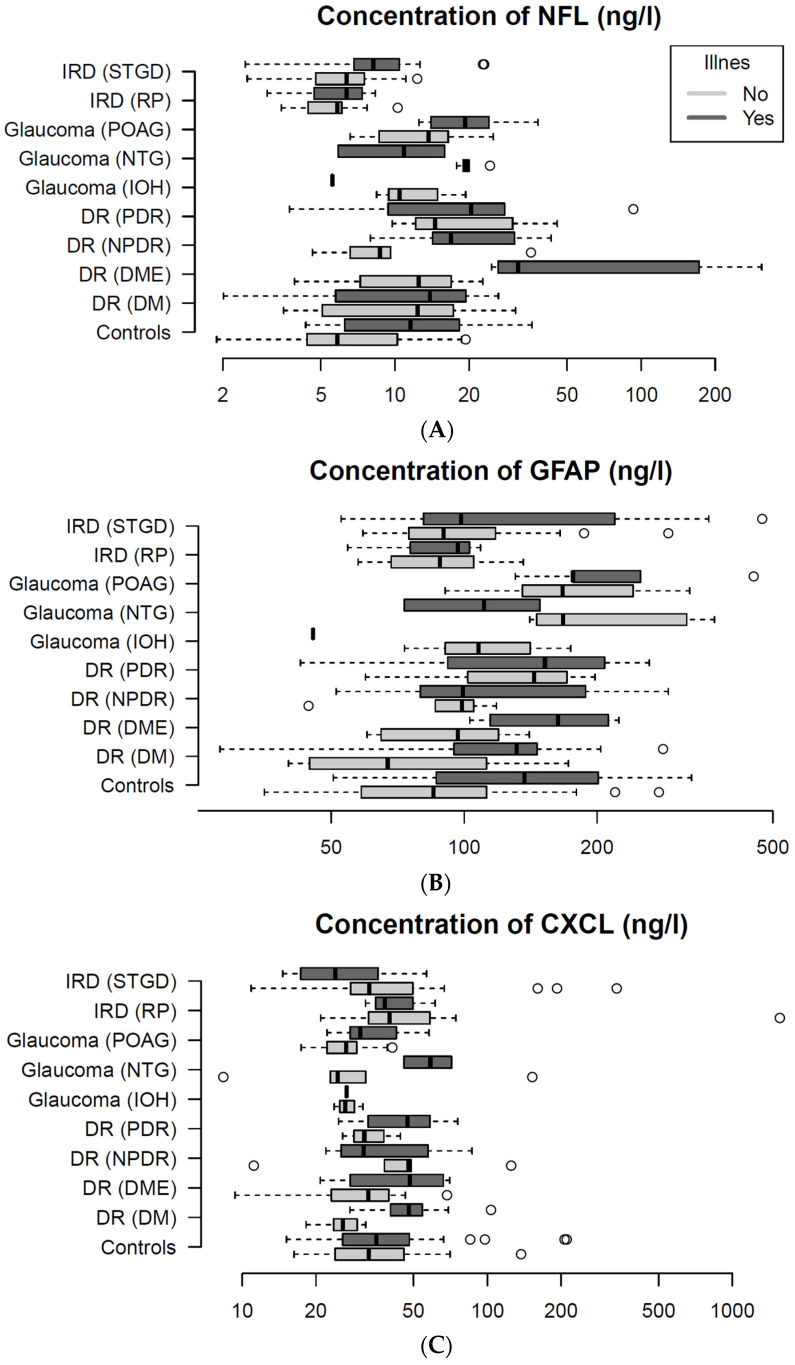
It presents plasma-levels of three biomarkers: Neurofilament Lights Chain (NFL; (**A**)), Glial Fibrillary Acidic Protein (GFAP; (**B**)), and C-X-C Motif Chemokine Ligand 13 (CXCL13; (**C**)) between disease sub groups. Plots presents plasma concentrations of the biomarker for the entire study population. (**A**) NFL levels vary dependent of disease subtype and presence of illness. The diabetes patients with diabetic macular edema have the highest NFL levels. Similarly, both (**B**) GFAP, and (**C**) CXCL13 concentrations also appear dependent on disease subtype and presence of illness. Circle (°) represents patients outliers.

In Figure 1, NFL, GFAP, and CXCL13 plasma concentrations are presented according to presence of illness for each disease subgroup. The biomarker concentrations vary depending on the type of biomarker, underlying retinal disease, and presence of comorbidities. Biomarker concentrations were generally higher in the ill subgroups.

**Figure 2:**
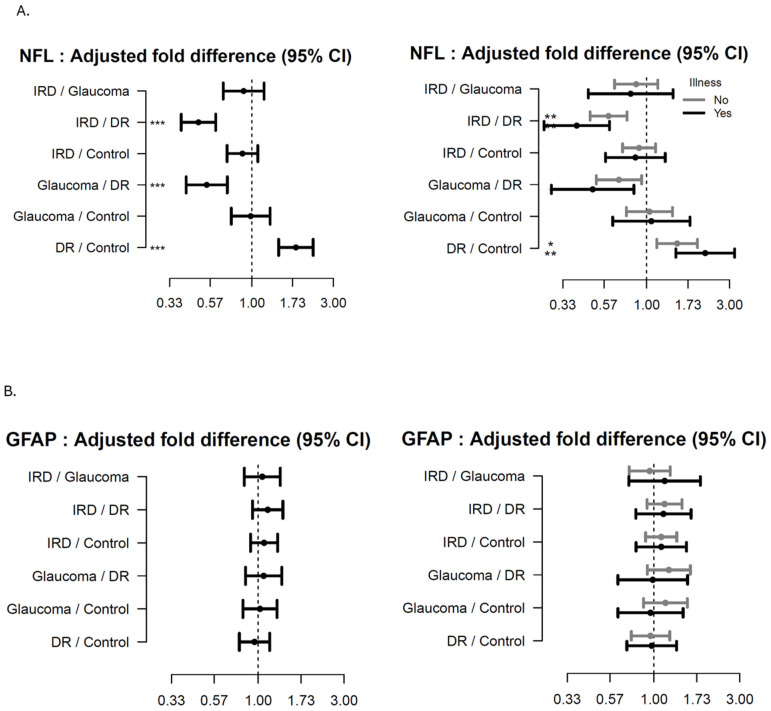

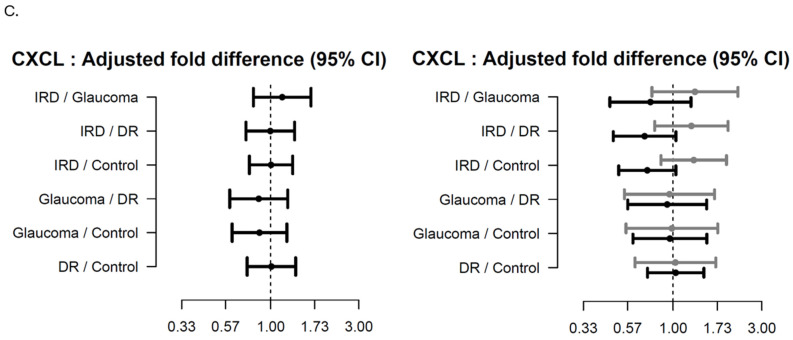
Forest plots showing the age-adjusted fold-difference in plasma-levels between the main disease groups with 95% confidence intervals (95% CI). The left panel shows the results of the primary analysis and the right panel the results of the corresponding subgroup analysis with participants stratified according to presence of illness. (**A**) Neurofilament Lights Chain (NFL), (**B**) Glial Fibrillary Acidic Protein (GFAP), (**C**) C-X-C Motif Chemokine Ligand 13 (CXCL13). adj-*p* < 0.1 * adj-*p* < 0.05, ** adj-*p* < 0.01, *** adj-*p* < 0.001.

As seen from Figure 2A–C (main groups) patients with IRD had decreased levels of NFL (age adjusted) compared to DR (FD 0.49 (95% CI 0.39;0.61, *p* < 0.001; adj-*p* < 0.001), independent of comorbidities. Patients with DR had increased levels of NFL (age-adjusted), compared to HCs (FD 1.81 95%CI: 1.43, 2.28, *p* < 0.001; adj-*p* < 0.001). See Supplementary S2 for further detail.

**Figure 3:**
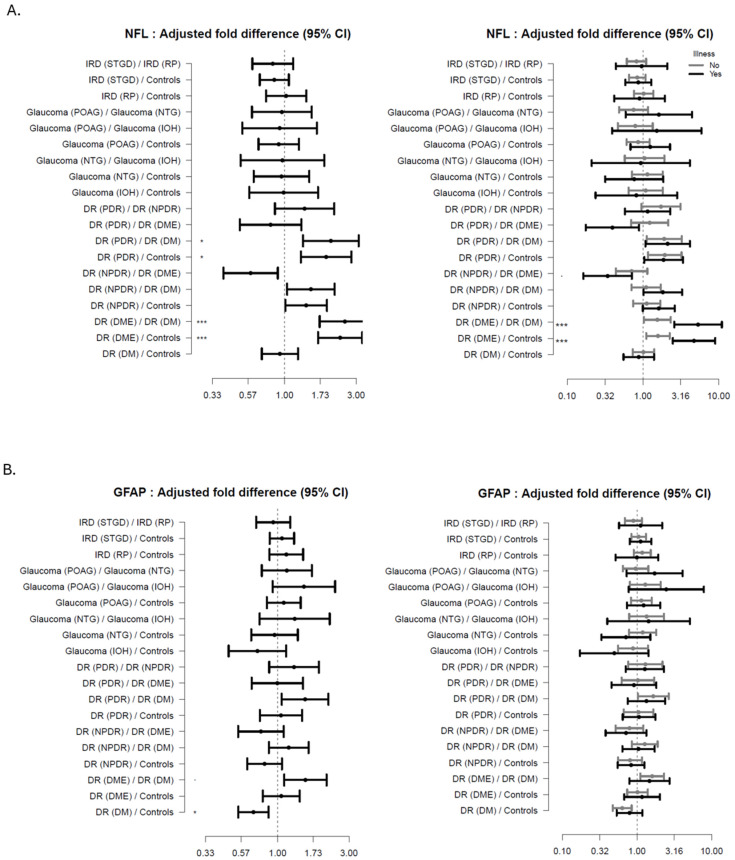

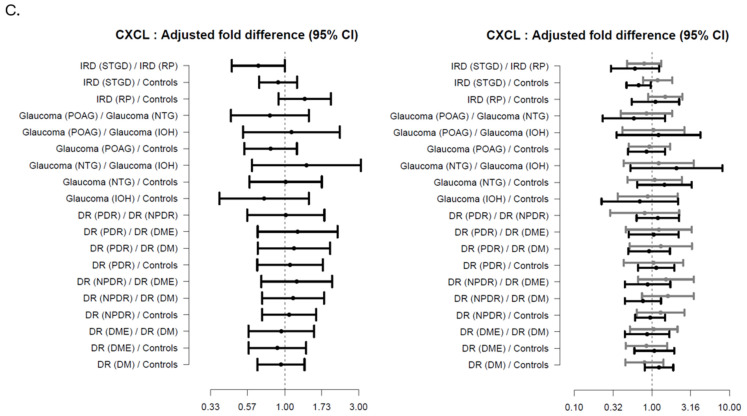
Forest plots showing the age-adjusted fold-difference in plasma-levels between the disease subgroups with 95% confidence intervals (95% CI). The left panel shows the results of the primary analysis and the right panel the results of the corresponding subgroup analysis with participants stratified according to presence of illness. (**A**) Neurofilament Lights Chain (NFL), (**B**) Glial Fibrillary Acidic Protein (GFAP), (**C**) C-X-C Motif Chemokine Ligand 13 (CXCL13). adj-*p* < 0.1 * adj-*p* < 0.05, *** adj-*p* < 0.001.

As seen from Figure 3A–C (subgroups) patients with advanced DR (PDR, DME) had increased levels of NFL (age-adjusted) compared to DM (FD 2.52 (95%CI: 1.71; 3.72, *p* < 0.001; adj-*p* < 0.001 and FD 2.04 (95%CI: 1.33; 3.12, *p* < 0.001; adj-*p* < 0.001), respectively) and HC (FD 2.35 (95%CI: 1.67; 3.30, *p* < 0.001; adj-*p* < 0.001), and FD 1.89 (95%CI: 1.28; 2.79, *p* < 0.001; adj-*p* < 0.001) respectively); In the same patients with no comorbidities a trend increase was found. Additionally, an increasing trend in NFL levels were found in NPDR compared to DM and HCs, and a decreasing trend was found in NPDR compared to DME participants.

For the patients having comorbidities, NFL levels were increased in DME compared to DM (FD 5.36 (95%CI: 2.60; 11.04, *p* < 0.001; adj-*p* < 0.001) and HC (FD 4.73 (95% CI: 2.48; 9.01, *p* < 0.001; adj-*p* < 0.001). In DR with comorbidities a graded trend increase in NFL levels was related to disease severity.

Patients with DM had decreased levels of GFAP (age-adjusted) compared to HC (FD 0.69; 95%CI 0.55, 0.87, *p* = 0.002, adj-*p* = 0.02), while a trend increase was seen in advanced DR compared to DM patients (±comorbidities).

A decreasing trend was seen in Stargaardt patients, compared to RP patients, along with Stargaardt patients compared to HCs, see Supplementary S3 for further detail.

## 3. Discussion

The primary aim of the study was to investigate whether the highly sensitive SiMoA-established biomarker for NFL, GFAP and CXCL13 (representing neurodegeneration and -inflammation) could be detected in the bloodstream of patients with retinal disease. We found that all biomarkers were detectable in the blood of all the individuals and, as expected, varied somewhat according to the presence of comorbidities [20,21]. This is important to consider before using these biomarkers in clinical practice [22]. Next, as per recent studies, we also found a strong association between both NFL and GFAP and age [23]. This is probably due to the continuous neurodegenerative progress occurring in body tissues as part of the aging process [7,24,25]. The lack of (or minor) association between CXCL13 and age could be due to its alternative role as a chemokine in B-cell associated immune responses.

The secondary aim was to explore the blood-biomarker concentrations in relation to each retinal disease. In this study we found significantly increased NFL blood-levels in advanced DR (i.e., PDR, DME), compared to DM and HCs. A similar trend increase was seen in DR patients without comorbidities. It suggests that our raised NFL plasma levels may be related to retinal damage involving the ganglion cells as part of DR development, independent of medical disorders [25]. In addition, the NFL expression appeared to be expressed in a dose-related manner, allowing detection of milder DR disease. These raised NFL levels could also be (at least in part) an expression of microvascular complications elsewhere [26]. Here larger datasets are needed to confirm these findings.

Few previous studies show NFL as a potential blood-biomarker for retinal diseases; nevertheless, its validity remains controversial. In preterm ROP infants, an increased NFL blood-level could also be related to brains insult during the neonatal period [27]. In addition, a recent study found NFL in corpus vitreum relating to Alzheimer’s disease and not retinal disease. Here NFL levels were related to amyloid beta, t-tau and selected inflammatory and vascular proteins instead [28]. In the case of patients with age-related macular degeneration, NFL was recently suggested to be dependent on age alone [24].

In this study, we found a decrease in GFAP-levels in DM patients compared to HCs. A previous study found similar results [29]. The authors showed increased GFAP blood-levels associated to older age, apolipoprotein epsilon4 status, and cognitive impairment, while decreased GFAP levels were associated with higher body mass index, diabetes, and tobacco use [29]. An increasing trend in GFAP levels were found in DR subgroups compared to DM. Analyzing GFAP levels in the main group of DR against DM (instead of HCs) would possibly have given us a larger disease response signal. This statement is largely supported by several previous studies suggesting that raised GFAP levels are in fact a ‘retinal stress response’ occurring in the Müller cells during disease progression [6,9,30]. Here, GFAP levels were elevated in the vitreous body of patients with retinal disease, and additionally in the anterior chamber liquid in DR patients [8,13,14]. Recently a case-control study based on data from the EUROCONDOR trial showed that GFAP-levels were higher at baseline in diabetes patients developing retinal neurodysfunction (as measured by multifocal electroretinography) two years later [7]. Summarizing, it seems that GFAP is not influenced by diabetes microvascular complications, as NFL might be, and therefore it stratifies DR from DM more clearly. It needs to be highlighted that GFAP levels seem to be more influenced by other medical comorbidities instead.

Regarding CXCL13, herein we found a trend increase in Stargardt patients compared to HCs; however, other medical comorbidities could also influence our results. To our knowledge, this is the first study to investigate such an association.

The three biomarkers appeared to distinguish between some of the retinal diseases having different main areas of the retina involved. NFL levels tended to be elevated in DR (involving inner retinal layers) compared to IRD (involving outer retinal layer), which could potentially suggest that NFL were released from the retinal ganglion cells into circulation during DR progression. Similarly, in glaucoma patients’ GFAP levels (coming from astrocytes and Müller cells trespassing the retina) were increased compared to DR and IRD patients (mainly involving inner or the outer retinal layer, respectively).

Finally, we wanted to explore the influence that other medical disorders may have on biomarker levels in the blood [21,22]. We found that the highest NFL levels were reached in patients having DME and comorbidities. This increase was probably mostly related to neurological diseases coexisting in this patient group. GFAP reached the highest levels in the glaucoma subgroups having comorbidities (POAG, NTG), probably resulting from the abundant disease-load often seen in older people. These results suggest that GFAP is less accurate to use as a blood-biomarker for retinal disease in patients suffering from (at least some) medical comorbidities.

### Strength and Limitations

The use of the ultrasensitive SiMoA platform is a great strength of this study, as it allowed us to detect our chosen biomarkers with great precision even in small quantities. Diabetes-related morbidities, such as hypertension and dyslipidemia, were common and could potentially influence our biomarker concentrations [21]. Although, as these conditions were generally medically well regulated, we do not consider this to have an impact on our study results.

There were some potential limitations to this study. Diabetic complications could potentially have influenced the biomarker levels. Patients generally had normal kidney function, so we do not consider diabetic nephropathy to have contributed to biomarker derangements [20,31]. In addition, in terms of periphery neuropathy, we found mostly normal clinical values (biothesiometry < 25) in diabetes patients (no comorbidities). Further, as none of the participants were diagnosed with either Alzheimer’s or other types of dementia, we do not expect this neurological complication to play a major role in our study findings [32,33,34].

In contrast, diabetes patients with comorbidities had abnormally high NFL values, suggesting either that these patients suffered from neurological diseases, or that such patients have aggravation in their diabetic complications as well.

## 4. Materials and Methods

This study is an exploratory, cross-sectional study of adult participants aged 19 to 75 years recruited from Copenhagen University Hospital at Rigshospitalet (period 2019 to 2020).

### 4.1. Identification and Recruitment of Participants

Study participants were recruited from the Department of Ophthalmology at Rigshospitalet. The study population consisted of 151 participants: 20 HC, and 131 with different chronic retinal degenerative diseases. The study population contained 131 participants with retinal disease divided into head-groups (and subgroups): 51 diabetes patients (13 non-proliferative retinopathy (NPDR), 8 proliferative retinopathy (PDR), and 11 diabetic macular edema (DME)), 27 glaucoma patients (16 primary open-angle glaucoma (POAG), 7 normal-tension glaucoma (NTG) and 4 intraocular hypertension (IOH)), 53 inherited retinal degeneration patients (16 retinitis pigmentosa (RP) and 37 Stargardt (STGD)) and 20 healthy controls (HCs). Individuals were labelled as having comorbidities if they had dysregulated organ function (liver, kidney parameters), active eye diseases or medical disorders considered to influence biomarker expression. Supplementary S1 presents the comorbidities for the disease subgroups.

### 4.2. Patient Data Retrieval and Definitions

Clinical information was retrieved from the electronic medical record system. We collected data on diagnosis, phenotype, age, gender, eye and systemic disease, medicine, and organ function (i.e., creatinine, glomerular filtration rate, alanine transaminase, alkaline phosphatase). Similarly, we gathered information from clinical eye examinations (i.e., visual acuity, fundus imaging, and intraocular pressure). For diabetes patients, the additional information gathered was disease duration, body mass index, blood pressure, pulse, biothesiometer (Biomedical Instrument Co, Newbury, OH, USA), and routine clinical follow-up blood parameters (i.e., hemoglobin, glycated-hemoglobin, glucose, total cholesterol, high-density lipoprotein, low-density lipoprotein, very-low-density protein, and triglycerides).

### 4.3. Sample Procedures, Target Analysis, and Measurements

Procedures were standardized according to the study protocol at the clinical biochemistry department at Rigshospitalet with blood samples were collected in standard tubes (MiniCollect^®^ K2EDTA, Greiner Bio-One, Kremsmünster, Austria), prepared and stored in a −80 °C freezer. At Vejle Hospital, commercially available assays for the Single molecule array (Simoa) HD-1 Analyzer (Quanterix©, Billerica, MA, USA) were used to quantify NFL (item 103186), GFAP (item 102336), and CXCL13 (item 102635) in plasma samples according to the manufacturer’s procedures. (3) The samples were diluted four-fold in buffer, included in the kits, and analyzed as single determinations. Three quality controls, two from kit material and one in-house prepared plasma pool were included in each run to evaluate assay performance. The total analytical coefficient of variations (CV%) was 7–13% (NFL), 5–16% (GFAP), and 7–19% (CXCL13).

### 4.4. Statistical Analysis

Descriptive statistics were reported as numbers (%) for categorical data and medians (quartiles) for quantitative data. Biomarker concentrations were visualized in boxplots. In the primary analysis, pairwise comparisons between study groups were made using ANCOVA adjusted for participants’ age. All outcomes were log-transformed prior to analysis due to substantial skewness. Hence, estimates are reported as fold differences (FD) with 95% confidence intervals. Goodness of fit was evaluated using residual diagnostics. Secondary subgroup analyses compared different diagnoses, severities, and phenotypes using an ANCOVA model similar to the primary analysis. The subgroup analyses were repeated with ‘ill’ subgroups. p-values were adjusted for multiple testing for primary and secondary analyses in turn using the method of Benjamini and Hochberg, which controls the false discovery rate [35]. An adjusted *p*-value < 0.05 was considered statistically significant. All analyses were performed with R statistical software version 4.4.2 [36].

## 5. Conclusions and Perspectives

These results imply that these biomarker-tests are useful for detecting and monitoring development of retinopathy in the circulations of diabetes patients, and perhaps also in patients with other chronic degenerative retinal diseases. Prospective studies are underway to examine this hypothesis in depth.

## Figures and Tables

**Table 1:** Clinical characteristics of study participants.

Variable	Diabetic Retinopathy (All)	Diabetic Retinopathy (−Comorbidity)	Diabetic Retinopathy (+Comorbidities)	Glaucoma (All)	Glaucoma (−Comorbidities)	Glaucoma (+Comorbidities)	Inherited retinal Degeneration (All)	Inherited Retinal Degeneration (−Comorbidities)	Inherited Retinal Degeneration (+Comorbidities)	HC (−Comorbidities)
Patients (n)	DM, NPDR, PDR, DME (19, 13, 8, 11)	DM, NPDR, PDR, DME (10, 5, 3, 7)	DM, NPDR, PDR, DME (9, 8, 5, 4)	HTG, NTG, IOH (16,7, 4)	HTG, NTG, IOH (11, 5, 3)	HTG, NTG, IOH (5, 2, 1)	RP, STGD (16, 37)	RP, STGD (13, 22)	RP, STGD (3, 15)	HCs (20)
Age	56.8 (46.3, 63.7)	51.7 (39.3, 59.9)	58.8 (53.7, 64.4)	73.7 (64.6, 78.7)	76.7 (64.6, 80.4)	71.0 (66.6, 73.7)	43.5 (33.1, 52.5)	39.2 (31.9, 50.5)	49.9 (38.7, 63.1)	34.1 (26.6, 59.9)
Gender (male)	28 (55%)	12 (48%)	16 (62%)	12 (44%)	10 (53%)	2 (25%)	30 (57%)	19 (54%)	11 (61%)	21 (45%)
Duration of diabetes (years)	19.0 (15.0, 23.0)	19.0 (15.2, 21.5)	23.0 (15.0, 31.0)	NA	NA	NA	NA	NA	NA	NA
VA (right)	1.0 (0.8, 1.0)	1.0 (0.8, 1.0)	1.0 (0.8, 1.0)	0.9 (0.6, 1.0)	1.0 (0.8, 1.0)	0.6 (0.4, 1.0)	0.1 (0.0, 0.6)	0.1 (0.0, 0.7)	0.1 (0.0, 0.2)	1.0 (1.0, 1.0)
VA (left)	1.0 (0.8, 1.0)	1.0 (0.8, 1.0)	0.9 (0.9, 0.9)	1.0 (0.6, 1.0)	1.0 (0.5, 1.0)	0.9 (0.7, 1.0)	1.0 (0.8, 1.0)	0.1 (0.0, 0.6)	0.1 (0.0, 0.2)	1.0 (1.0, 1.0)
IOP (right)	NA	NA	NA	13.0 (11.2, 22.5)	13.0 (10.5, 15.0)	18.5 (11.8, 25.2)	1.0 (0.5, 1.0)	15.0 (10.0, 17.0)	18.0 (14.0, 19.0)	15.0 (14.5, 15.8)
IOP (left)	NA	NA	NA	13.0 (11.0, 16.5)	13.0 (10.2, 16.5)	12.5 (11.8, 14.5)	15.0 (12.0, 17.0)	14.0 (11.0, 16.0)	17.0 (12.0, 19.0)	16.5 (15.5, 17.2)
Macular OCT (right, microns)	NA	NA	NA	NA	NA	NA	204.0 (177.5, 238.5)	210.0 (176.5, 261.0)	202.0 (183.2, 220.5)	266.0 (266.0, 266.0)
Macular OCT (left, microns)	NA	NA	NA	NA	NA	NA	209.0 (170.0, 247.5)	213.0 (166.0, 256.5)	194.0 (175.8, 220.0)	271.0 (271.0, 271.0)
* Papillary OCT (right, microns)	NA	NA	NA	53.5 (43.0, 61.8)	53.5 (43.2, 61.5)	52.5 (42.2, 63.5)	NA	NA	NA	NA
* Papillary OCT (left, microns)	NA	NA	NA	50.5 (46.2, 63.0)	49.5 (46.2, 60.5)	58.0 (49.8, 64.8)	NA	NA	NA	NA
* GCL thickness (right, mm)	NA	NA	NA	0.7 (0.6, 0.9)	0.8 (0.7, 0.9)	0.6 (0.5, 0.8)	NA	NA	NA	NA
* GCL thickness (left, mm)	NA	NA	NA	0.7 (0.7, 0.9)	0.7 (0.7, 0.9)	0.7 (0.6, 0.8)	NA	NA	NA	NA
VFD (right)	NA	NA	NA	9.6 (6.8, 20.0)	9.0 (6.6, 19.4)	11.8 (9.0, 21.2)	NA	NA	NA	NA
VFD (left)	NA	NA	NA	10.1 (4.9, 17.4)	14.6 (3.6, 18.4)	7.6 (6.2, 9.9)	NA	NA	NA	NA
BP (systolic; mmHg)	135.0 (127.0, 139.0)	130.5 (127.0, 137.5)	149.0 (135.0, 151.0)	NA	NA	NA	NA	NA	NA	NA
BP (diastolic; mmHg)	78.0 (70.0, 84.0)	74.0 (70.5, 83.0)	83.0 (75.0, 93.0)	NA	NA	NA	NA	NA	NA	NA
Pulse (bpm)	78.0 (72.0, 86.0)	77.0 (71.5, 85.2)	78.0 (74.0, 86.0)	NA	NA	NA	NA	NA	NA	NA
Biothesiometry (right)	23.5 (14.5, 45.0)	18.0 (12.0, 28.0)	40.0 (18.5, 47.5)	NA	NA	NA	NA	NA	NA	NA
Biothesiometry (left)	22.0 (14.8, 42.0)	17.0 (12.0, 28.0)	30.0 (18.5, 44.0)	NA	NA	NA	NA	NA	NA	NA
Weight (kg)	82.7 (72.0, 96.6)	82.1 (77.3, 87.2)	91.4 (68.3, 111.4)	NA	NA	NA	NA	NA	NA	NA
Height (cm)	168.0 (164.0, 181.0)	170.5 (161.0, 184.8)	165.0 (164.0, 171.0)	NA	NA	NA	NA	NA	NA	NA
BMI (kg/m^2^)	28.3 (26.6, 31.0)	28.0 (26.7, 30.0)	28.8 (26.5, 38.0)	NA	NA	NA	NA	NA	NA	NA
Haemoglobin (mmol/L)	9.1 (7.9, 9.5)	9.1 (8.6, 9.4)	8.4 (7.6, 9.4)	8.9 (8.5; 9.2)	8.9 (8.5, 9.2)	8.7 (8.3, 9.1	8.6 (8.2, 9.1)	8.7 (8.35, 9.2)	8.4 (8.0, 8, 9)	9.0 (8.8, 9.2)
Creatinine (µmol/L)	68.0 (59.5, 81.8)	62.0 (57.5, 75.0)	84.0 (72.5, 101.5)	77 (68.0, 81.0)	77.0 (68.0, 81.5)	80.0 (74.5, 94.2)	71 (64.0, 84.0)	72.0 (66.5, 83.5)	70.5 (6.3, 72.8)	63.5 (59.5, 73.8)
eGFR (mL/min)	60.0 (60.0, 60.0)	60.0 (60.0, 60.0)	60.0 (55.5, 60.0)	80.0 (67.0, 87.5)	73.0, (75.0, 88.0)	69.0 (62.5, 84.0)	90.0 (89.0, 90.0)	90.0 (89.0, 90.0)	90.0 (84.3, 90.0)	60.0 (60.0, 60.0)
ALT (U/L)	29.0 (21.8, 34.5)	29.0 (21.0, 34.0)	29.0 (26.0, 33.5)	21.0 (16.5, 26.0)	20.0 (15.5, 24.5)	24.0 (20.0, 31.0)	22.0 (18.0, 30.00)	21.0 (18.0, 29.5)	23.5 (19.5, 31.8)	23.0 (18.2, 29.2)
ALP (U/L)	88.0 (74.0, 95.5)	88.0 (74.0, 109.0)	88.0 (85.0, 89.0)	71.0 (54.0, 89.0)	64.0 (49.8, 85.8)	88.0 (88.0, 93.0)	67.0 (58.0, 84,0)	64.0 (54.0, 84.0)	71.0 (64.5, 83.2)	60.0 (54.2, 62.0)
HbA1c (mmol/mol)	62.0 (51.0, 82.0)	62.0 (52.2, 79.5)	67.0 (54.0, 80.5)	NA	NA	NA	NA	NA	NA	63.5 (60.0, 69.5)
Glucose (mmol/L)	9.9 (8.3, 12.7)	9.9 (8.5, 12.3)	10.6 (8.8, 12.6)	NA	NA	NA	NA	NA	NA	9.0 (9.0, 9.0)
Cholesterol (mmol/L)	4.1 (3.7, 4.7)	4.4 (4.0, 4.9)	3.7 (3.5, 4.2)	NA	NA	NA	NA	NA	NA	2.4 (1.9, 2.9)
HDL (mmol/L)	1.2 (0.9, 1.7)	1.2 (1.0, 1.5)	0.9 (0.8, 1.8)	NA	NA	NA	NA	NA	NA	NA
LDL (mmol/L)	2.2 (1.9, 2.8)	2.2 (2.0, 2.8)	1.9 (1.3, 2.5)	NA	NA	NA	NA	NA	NA	NA
VLDL (mmol/L)	0.6 (0.4, 1.0)	0.5 (0.4, 1.0)	0.9 (0.7, 1.0)	NA	NA	NA	NA	NA	NA	NA
TG (mmol/L)	1.5 (1.1, 2.5)	1.2 (0.9, 2.8)	2.1 (1.8, 2.4)	NA	NA	NA	NA	NA	NA	NA

Median (IQR) were presented for continuous variables, and N (%) was presented for categorical variables. Abbreviations: visual acuity, VA; body mass index, Intraocular Pressure, IOP; ganglion cell layer, GCL; visual field defect, VFD; Blood pressure, BP; Beats per minute, BPM; Body mass index, BMI; Glomerular filtration rate, eGFR; Alanine aminotransferase, ALT; Alkaline phosphatase, ALP; High-density lipoprotein, HDL; Low-density lipoprotein, LDL; Very-low-density protein, VLDL; triglycerides, TG. * Heidelberg, glaucoma module: Normal RNFL thickness around 100 microns. Normal GCL thickness is around 24.0 microns.

## Data Availability

The datasets used and/or analyzed during the current study are available from the corresponding author on reasonable request.

## Supplementary Materials

The following supporting information can be downloaded at: https://www.mdpi.com/article/10.3390/ijms26062625/s1.
